# Determination of Glycidol in Soy Sauce Using p-Dimethylaminophenol Derivatization Coupled with Liquid Chromatography-Tandem Mass Spectrometry

**DOI:** 10.3390/foods15071220

**Published:** 2026-04-03

**Authors:** Yifan Zhao, Peng Wang, Longlong Wang, Lixia Qin, Hai Chi, Guangxin Yang, Xiaosheng Shen, Chengqi Fan, Xiaoqing Tian, Mian Hasnain Nawaz, Cong Kong

**Affiliations:** 1 East China Sea Fisheries Research Institute, Chinese Academy of Fishery Sciences, Shanghai 200090, China; yifanzhao993@163.com (Y.Z.); wptc546092937@163.com (P.W.); longlongwang2022@163.com (L.W.); foodsmc98@126.com (X.S.); fancq@eastfishery.ac.cn (C.F.); amytian0904@126.com (X.T.); 2 College of Food Science and Engineering, Dalian Ocean University, Dalian 116034, China; 3 School of Chemical and Environmental Engineering, Shanghai Institute of Technology, 100 Haiquan Road, Shanghai 201418, China; lxqin@sit.edu.cn; 4 Key Laboratory of Protection and Utilization of Aquatic Germplasm Resource, Liaoning Ocean and Fisheries Science Research Institute, Dalian 116023, China; andychihai@126.com; 5 Interdisciplinary Research Centre in Biomedical Materials (IRCBM), COMSATS University Islamabad, Lahore Campus, Lahore 54000, Pakistan; mhnawaz@cuilahore.edu.pk

**Keywords:** glycidol, soy sauce, p-Dimethylaminophenol, derivatization, carbon yarn, HPLC-MS/MS, food contaminant

## Abstract

Glycidol, a probable human carcinogen, remains an under-investigated process contaminant in soy sauce. This study developed a sensitive and specific liquid chromatography-tandem mass spectrometry (LC-MS/MS) method for its determination in this complex condiment. The approach combined chemical derivatization with p-Dimethylaminophenol hydrochloride for analyte stabilization with an optimized sample pretreatment using a custom-packed activated carbon solid-phase extraction (SPE) cartridge effectively removed matrix interferences, and performing the derivatization at pH 6.5 prevented conversion of 2- and 3-monochloropropanediol (2-MCPD and 3-MCPD) into glycidol, ensuring high specificity and accuracy. This approach shows broad linearity from 1 to at least 100 ng/mL (R^2^ = 0.9993), and demonstrates excellent performance, with a limit of detection and quantification of 0.5 ng/mL and 1.0 ng/mL, respectively. Application to commercial samples (*n* = 11) confirmed the presence of glycidol, highlighting the need for its monitoring. This work provides a robust analytical tool essential for supporting food safety surveillance of this contaminant in fermented foods.

## 1. Introduction

Glycidol is a process-induced contaminant that forms predominantly during high-temperature food processing, especially when fats and oils undergo thermal degradation [1,2,3]. It has been documented in refined edible oils and other heat-treated foods, often in the form of fatty acid esters known as glycidyl esters (GEs) [4]. Owing to its genotoxic and carcinogenic properties, glycidol is classified by the International Agency for Research on Cancer (IARC) as a Group 2A probable human carcinogen [5,6]. In response to the health risks of even low-level exposure, food safety authorities including the European Food Safety Authority (EFSA) have implemented maximum levels for glycidyl esters in specific food commodities [7], thereby reducing dietary exposure to this processing contaminant.

Soy sauce is an extensively consumed condiment worldwide, particularly central to Asian cuisines, and is used as a key ingredient in a wide variety of dishes [8,9]. Its production typically involves fermentation followed by thermal processing (such as pasteurization or concentration), conditions under which contaminants like glycidol could potentially form [10]. Given the high consumption of soy sauce, even trace levels of glycidol in this condiment may contribute to significant cumulative dietary exposure over time. Thus, the ability to accurately detect and quantify glycidol in soy sauce is essential for protecting consumers and for meeting regulatory standards aimed at limiting carcinogenic contaminants in foods [11].

In food products, glycidol contamination can exist in two forms, as free glycidol or bound in glycerol-based fatty acid esters (i.e., GEs) [7,12]. Analytical methods often target the total glycidol content by hydrolyzing GEs to release free glycidol, which is then measured as an equivalent of glycidyl ester contamination [13]. This approach has been widely applied to fatty matrices such as vegetable oils and oil-rich foods [14,15]. By contrast, free glycidol in non-fatty fermented condiments (e.g., soy sauce) remains poorly characterized, with no validated method specifically developed for this matrix [5,16]. Developing a glycidol detection method specifically tailored to soy sauce is, therefore, critical for food safety assurance and regulatory compliance in this context. While the individual analytical techniques employed in this study, such as derivatization and SPE cleanup, have been reported in our previous research, their systematic application and validation for free glycidol in soy sauce has not been previously demonstrated. The acidic, fermented nature of soy sauce may facilitate the hydrolysis of GEs, and diverse modern production processes increase the potential for unintended formation. Thus, this study establishes the first validated method for determining free glycidol in soy sauce, addressing a critical data gap for this specific food matrix.

Accurate analysis of glycidol in soy sauce poses several analytical challenges. First, glycidol lacks a strong chromophore, which complicates direct detection by conventional high-performance liquid chromatography-ultraviolet (HPLC-UV) or fluorescence methods [17,18]. Second, the epoxide structure of glycidol is highly reactive and unstable, making the compound prone to degradation or reacting with other matrix constituents, thereby compromising direct quantification. Third, the complex matrix of soy sauce (containing a multitude of amino acids, peptides, sugars, and pigments) can introduce severe matrix interferences that hinder sensitivity and accuracy [19,20]. To overcome these obstacles, chemical derivatization is commonly employed to convert glycidol into a more stable and easily detectable derivative [21].

Existing analytical protocols for glycidol and related process contaminants often rely on gas chromatography–mass spectrometry (GC-MS) after derivatization [22,23,24]. For instance, Zhang et al. developed a GC-MS/MS method for analyzing 2- and 3-monochloropropanediol esters (2/3-MCPDEs) and glycidyl esters in milk powder [25]. Similarly, Wang et al. applied a GC-MS approach for these contaminants in vegetable oils [26]. While GC-MS based methods have been useful, they can be time-consuming and often face challenges with sensitivity and matrix contamination and require labor-intensive sample preparation and derivatization steps. Notably, interference from structurally similar compounds such as 2-monochloropropane-1,3-diol (2-MCPD) and 3-monochloropropane-1,2-diol (3-MCPD) can complicate glycidol analysis. Under strongly alkaline conditions (commonly used in certain derivatization or hydrolysis procedures), 3-MCPD can convert into glycidol [13,27,28], risking overestimation of glycidol if not carefully controlled. These limitations underscore the necessity for a matrix-appropriate analytical method specifically designed for soy sauce, rather than simply adapting protocols developed for other food matrices.

In this context, liquid chromatography-tandem mass spectrometry (LC-MS/MS) offers significant advantages for glycidol analysis in complex foods. However, to date, no validated LC-MS/MS method has been reported specifically for the determination of free glycidol in soy sauce. To address this knowledge gap, we present a derivatization-enhanced LC-MS/MS method that selectively targets free glycidol without hydrolysis from its ester form, while avoiding interference from related contaminants. The method employs p-Dimethylaminophenol (DMAphenol) as the derivatization reagent to rapidly react with the epoxide group of glycidol under mild conditions, forming a stable derivative compatible with LC-MS/MS detection. While derivatization with DMAphenol has been previously reported, its application to soy sauce required extensive matrix-specific optimization, including systematic evaluation of SPE sorbents, precise pH control to prevent 3-MCPD conversion, and comprehensive validation of performance parameters in this complex matrix. By optimizing the derivatization conditions (particularly the reaction pH to suppress 2/3-MCPD conversion) and incorporating an effective matrix cleanup step, we aimed to achieve trace-level sensitivity and high accuracy in glycidol quantification. We then validated the method in terms of sensitivity, linearity, precision, and recovery, and applied it to commercial soy sauce products. The resulting method is intended as a practical tool for routine food safety monitoring and regulatory compliance, ultimately helping to address the challenge in glycidol surveillance for non-fatty food products and to protect consumers from this toxic contaminant.

## 2. Materials and Methods

### 2.1. Reagents and Chemicals

All reagents and solvents were of analytical or HPLC grade. Glycidol and its isotopically labeled analog glycidol-d5 were purchased from Dr. Ehrenstorfer GmbH (Augsburg, Germany) with a purity of ≥99%. 3-MCPD, 2-MCPD, 3-MCPD-d5 and 2-MCPD-d5 (all purity >99%) were also from Dr. Ehrenstorfer. Ethanol, ethyl acetate, p-Dimethylaminophenol hydrochloride (DMAphenol, derivatization reagent), sodium hydroxide (NaOH), hydrochloric acid (HCl), and anhydrous sodium sulfate (Na_2_SO_4_) were purchased from Shanghai Acmec Biochemical Co., Ltd. (Shanghai, China). HPLC-grade methanol (MeOH), acetonitrile, formic acid, and ammonium acetate were obtained from Merck (Darmstadt, Germany). Poly(tetrafluoroethylene) (PTFE) syringe filters (0.22 μm pore size) were supplied by Tianjin Branch Billion Lung Experimental Equipment Co., Ltd. (Tianjin, China). Solid sorbents used for sample pretreatment included medicinal-grade activated carbon (approx. 200 mesh, composed of carbon yarn), ODS C18 (octadecylsilane), a graphitized carbon black (PestCarb-GCB) and empty 6 mL solid-phase extraction (SPE) cartridges with hydrophilic polyethylene frits (both from Adamas, Shanghai, China). Deionized water (18.2 MΩ·cm resistivity) was produced using a Milli-Q purification system (Millipore, Bedford, MA, USA).

For SPE cartridge preparation: A self-packed SPE cartridge (for single use) was prepared by loading 0.6 g of activated charcoal (Shanghai Titan Scientific Co., Ltd., Shanghai, China) into an empty SPE column. Prior to use, the column was conditioned by eluting 5 mL of MeOH followed by 5 mL of water to ensure optimal purification performance.

Standards and working solutions: Single stock solutions of glycidol (1 mg/mL) and glycidol-d5 (2 mg/mL) were prepared in acetonitrile, with glycidol-d5 serving as the isotopically labeled internal standard for glycidol. Working solutions were prepared by appropriately diluting the stock solutions with MeOH. All standard solutions were stored in amber glass vials at −20 °C, protected from light.

Derivatization Reagent: DMAphenol hydrochloride (100 mg/mL in deionized water), sodium hydroxide solution (4 M NaOH), and phosphate buffer (PB buffer, pH 6.5, prepared by mixing 31.5 mL of 0.2 M NaH_2_PO_4_ and 68.5 mL of 0.2 M Na_2_HPO_4_) were stored at 4 °C. Standards of 3-MCPD and its deuterated compound (3-MCPD-d5) were prepared as 1 µg/mL working solutions in MeOH and stored at −20 °C in amber vials. To prevent degradation, all prepared standards and working solutions were stored in the dark.

### 2.2. Sample Collection

The collected commercial samples encompassed both major categories of soy sauce: light (Sheng Chou) and dark (Lao Chou) types. It is noted that the dark soy sauce undergoes prolonged aging and may contain added caramel, resulting in a darker, thicker product, which contributes to a more complex matrix.

Eleven soy sauce samples (various brands and batches) were collected from local retail markets in Shanghai between November and December 2022, representing nine different manufacturers. The selection included a broad range of brands and production batches, encompassing both light and dark soy sauce varieties. To ensure representativeness and minimize sampling bias, the products were randomly purchased from different outlets and kept in their original sealed packaging during transport at ambient temperature. In the laboratory, each sample was thoroughly homogenized using a vortex mixer (WH-2 model) to achieve a uniform matrix. After homogenization, aliquots of each soy sauce were transferred into clean amber glass bottles with airtight caps to minimize light-induced degradation. All commercial soy sauce samples were analyzed within their labeled shelf-life periods as indicated by the manufacturers, ensuring sample integrity during storage prior to analysis.

### 2.3. Sample Preparation

An aliquot of 5 mL of homogenized soy sauce was transferred into a 15 mL polypropylene centrifuge tube. Dark soy sauces were diluted 5-fold to 10-fold before analyzing to increase fluidity. A volume of 100 μL of an internal standard working solution (glycidol-d5, 500 ng/mL in MeOH) was added to the sample, and the tube was manually shaken for 1 min to mix the contents thoroughly. The spiked sample was then loaded onto a pre-conditioned activated carbon SPE cartridge (6 mL format) that had been conditioned with 5 mL of MeOH followed by 5 mL of deionized water. The initial effluent (approximately 1–2 mL) was discarded to remove any impurities not retained by the sorbent. Glycidol was subsequently eluted from the cartridge using 2 mL of a 40% (*v*/*v*) MeOH–water solution, and the eluate was collected in a clean tube. The collected eluate was passed through a 0.22 μm PTFE membrane filter to remove any remaining particulate matter. Finally, the filtered extract (0.5 mL) was diluted 1:1 (*v*/*v*) with 0.1 M PB buffer (pH 6.5). The resulting mixture, with a total volume of approximately 1 mL, was transferred into a sample vial and served as the final solution ready for the derivatization reaction.

### 2.4. Derivatization

A 0.5 mL aliquot of the post-SPE purified and diluted sample extract was transferred to a glass autosampler vial, followed by addition of 50 μL DMAphenol derivatization reagent (100 mg/mL). The vial was sealed and manually vortexed for 1 min to ensure homogenization. The mixture was equilibrated at room temperature (22 °C) for 30 min, revortexed for 1 min, and subsequently incubated at 60 °C for 4 h to facilitate complete derivatization, converting glycidol to its DMAphenol derivative. After derivatization, samples were cooled to ambient temperature and directly subjected to LC-MS/MS analysis without further treatment.

### 2.5. LC-MS/MS Conditions

Derivatized samples were analyzed using an UltiMate 3000 UHPLC system coupled to a Q Exactive™ quadrupole-Orbitrap mass spectrometer (Thermo Fisher Scientific, Waltham, MA, USA). Chromatographic separation was performed on an Eclipse Plus C18 RRHD column (3.0 × 150 mm, 1.8 μm particle size; Agilent) maintained at 35 °C with a 0.5 mL/min flow rate. The mobile phase comprised: (A) 0.2% formic acid and 5 mM ammonium acetate in water and (B) 0.2% formic acid in MeOH. Gradient elution proceeded as follows: 0–1.5 min, 1% B; 1.5–6.0 min, 20% B; 6.0–6.15 min, 100% B; 6.15–8.5 min, 100% B; 8.5–8.65 min, 1% B; 8.65–10 min, 1% B (post-run equilibration). Injection volume was 4 μL.

The mass spectrometer was equipped with a heated electrospray ionization (HESI) source operating in positive ion mode. The HESI source parameters were as follows: spray voltage +3.5 kV; sheath gas flow rate 50 (arb units); auxiliary gas flow rate 15 (arb units); capillary temperature 350 °C. Data acquisition was carried out in full scan mode combined with parallel reaction monitoring (PRM) for the target analytes. The Orbitrap analyzer was set to a resolving power of 35,000 (at *m*/*z* 200), with an automatic gain control (AGC) target value of 1 × 10^6^ ions and a maximum injection time of 100 ms.

For identification and quantification of the derivatized glycidol, the exact mass of the protonated derivative and its fragment ions were monitored. The derivatized glycidol (glycidol-DMAphenol adduct) showed a [M + H]^+^ ion at *m*/*z* 212.1277. In PRM scans, this precursor ion was selected and fragmented to produce characteristic product ions at *m*/*z* 137.0833 and *m*/*z* 136.0755, corresponding to fragments of the derivatizing reagent moiety. The isotopically labeled internal standard (derivatized glycidol-d5) exhibited a [M + H]^+^ at *m*/*z* 217.1584 (as expected, 5 Da higher), and produced the same major fragment ion at *m*/*z* 137.0833. The retention time of the glycidol derivative under these conditions was approximately 2.60 min, with the internal standard co-eluting, confirming that both labeled and unlabeled glycidol derivatives chromatograph similarly. MS/MS parameters and the monitored fragments for 3-MCPD and 3-MCPD-d5 are outlined in Table 1. All operations, data acquisition, and analysis were conducted using Thermo Xcalibur 3.1 software.

### 2.6. Method Validation

The analytical method was validated with respect to linearity, sensitivity, accuracy, and precision using spiked soy sauce samples. Calibration standards were prepared by spiking in 40% methanol solvent with glycidol at concentrations of 1, 2, 5, 10, 20, 50, and 100 ng/mL, respectively, and glycidol-d5 at 10 ng/mL. They were derivatized as described in Section 2.4 before LC-MS/MS analysis. Calibration curves were constructed by plotting the peak area ratio of glycidol (derivative) to the internal standard versus the known glycidol concentration. A linear least-squares regression was used to evaluate linearity. Sensitivity was assessed by determining the limits of detection (LOD) and quantification (LOQ). The LOD and LOQ for glycidol in soy sauce were estimated based on signal-to-noise (S/N) criteria of approximately 3:1 and 10:1, respectively, using low-concentration spiked samples.

Accuracy and intra-day precision were assessed through recovery experiments at three concentration levels. Blank soy sauce samples were spiked with glycidol at 5 ng/mL (low), 20 ng/mL (medium), and 40 ng/mL (high), in triplicate for each level on the same day. After processing and analysis, the measured glycidol concentrations were compared to the spiked values to calculate the percentage recovery. The relative standard deviation (RSD) of the recoveries at each level provided a measure of intra-day precision (repeatability). To evaluate inter-day precision (reproducibility), the 20 ng/mL spiked recovery experiment was repeated on three different days (again in triplicate each day), and the RSD across these days was calculated.

## 3. Results

### 3.1. Characterization of Reaction Product of Glycidol and p-Dimethylaminophenol

**Figure 1:**
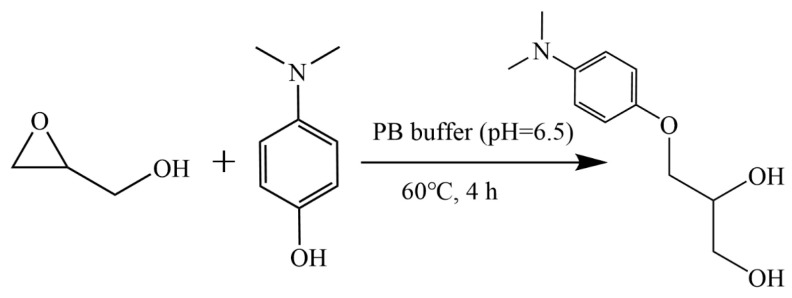
Scheme of glycidol derivatization reaction with DMAphenol.

Glycidol in its native form is challenging to detect and quantify due to its chemical characteristics. It has no strong chromophore for optical detection and exhibits low ionization efficiency in electrospray mass spectrometry, partly because it lacks readily ionizable functional groups. Moreover, its epoxide ring structure is susceptible to hydrolysis and other side reactions, leading to potential loss of the analyte. Attempts to sensitively detect underivatized glycidol in soy sauce were unsuccessful, confirming the necessity of derivatization. To address these issues, we introduced a derivatization step using DMAphenol hydrochloride, which reacts specifically with glycidol to form a more stable derivative (in Figure 1). DMAphenol was selected as the derivatizing agent due to its capacity for epoxide ring cleavage under mild conditions (60 °C) and formation of analytically stable adducts. The derivatization significantly improves the detectability of target analyte: the resulting derivative has higher polarity and a UV-active aromatic moiety, facilitating chromatographic separation, and yields distinct fragment ions that enhance mass spectrometric identification and quantification [28].

**Figure 2:**
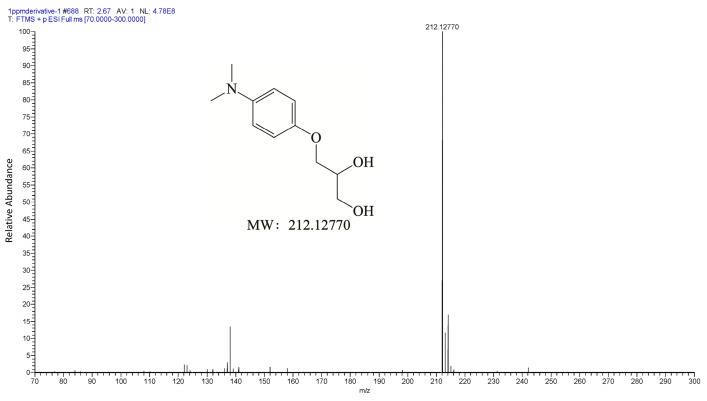
High-resolution mass spectrum of the derivatized product of glycidol with DMAphenol (1 mg/mL, in MeOH, electrospray ionization).

We verified the structure and properties of the glycidol derivative through spectral characterization. In the LC-MS/MS analysis, derivatized glycidol showed an exact protonated molecular ion at *m*/*z* 212.13, matching the theoretical [M + H]^+^ of the expected derivative (Figure 2 and Figure S1). Tandem MS fragmentation of the precursor ion yielded diagnostic fragments at *m*/*z* 137 and 136, corresponding to the dimethylaminophenol moiety, confirming successful epoxide ring derivatization of glycidol. Similarly, the derivatized internal standard (glycidol-d5) gave an [M + H]^+^ at *m*/*z* 217.13 (Figure S1), exactly 5 Da higher, and yielded the same fragment ions, confirming identical derivatization efficiency for the isotopically labeled analog. Furthermore, chromatographic analysis of blank matrix samples after derivatization showed clean baselines with no interfering peaks at the retention time of the glycidol-DMAphenol derivative, and no additional peaks corresponding to potential structurally similar compounds were observed (Figure S2), confirming that no analogous derivatives were formed under the optimized conditions. Additionally, nuclear magnetic resonance (NMR) spectroscopy was employed to confirm the structure of derivative (Figures S3 and S4). The ^13^C NMR and ^1^H NMR spectra showed chemical shifts consistent with the proposed structure of the glycidol-DMAphenol adduct, including signals for aromatic carbons/protons and the glycerol backbone, thereby definitively confirming the identity of the derivative. Together, these results demonstrate that DMAphenol hydrochloride reacts efficiently and selectively with glycidol, yielding a stable derivative suitable for sensitive analysis in a complex matrix. A recently reported method by Wang et al. [29] used HFBI derivatization with GC-MS for the determination of glycidol, 3-MCPD, and 2-MCPD in heated tobacco product aerosols. Their approach achieved multi-analyte detection in a relatively simple matrix. In contrast, our method is specifically designed for the complex soy sauce matrix. We employ DMAphenol derivatization with LC-MS/MS, which is performed directly in the aqueous phase, providing higher sensitivity (LOQ 1.0 ng/mL) and simplified sample handling.

### 3.2. Optimization of Derivatization Conditions

**Figure 3:**
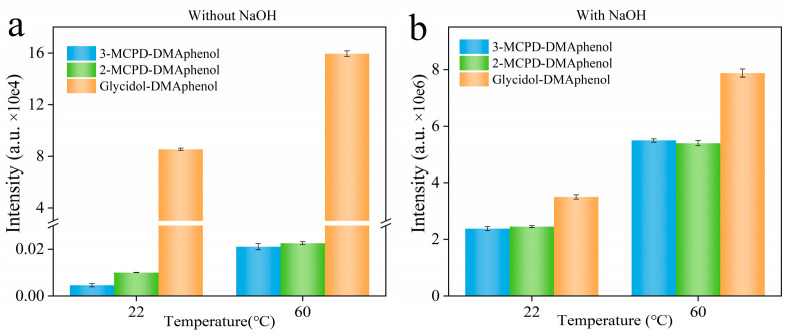
Signal response of derivatized 3-MCPD, 2-MCPD, and glycidol under alkaline vs. neutral conditions: (**a**) with 4 M NaOH (50 μL), (**b**) without NaOH. Analyte concentration: 100 ng/mL each. The break symbols in the y-axis of (**a**) indicate a discontinuity in the scale. This design is adopted to simultaneously visualize the low-response data of MCPD derivatives and the high-response data of glycidol derivative in the same plot, ensuring the visual clarity of both datasets is not compromised.

One critical parameter for the derivatization reaction is the solution pH. Previous studies have shown that under strongly alkaline conditions, 3-MCPD can convert to glycidol [28], which can undergo dehydrochlorination, where the chlorine atom is eliminated together with a hydrogen atom from an adjacent hydroxyl group, leading to the formation of an epoxide ring—i.e., glycidol. This reaction is particularly relevant during derivatization or hydrolysis steps if the pH is not carefully controlled, as it may lead to overestimation of glycidol content in samples containing 3-MCPD. Given that soy sauce may contain 3-MCPD as a process contaminant, strongly alkaline derivatization conditions could promote glycidol formation from 3-MCPD, resulting in erroneous glycidol quantification. To mitigate this interference, we systematically investigated pH effects on derivatization selectivity toward glycidol. Equimolar model solutions of 2-MCPD, 3-MCPD, and glycidol were treated with derivatization reagent under systematically varied conditions to assess reaction specificity: comparative derivatization was performed with and without 4 M NaOH supplementation at both ambient temperature (22 °C) and 60 °C. The result (Figure 3) demonstrates that alkaline conditions (4 M NaOH) significantly enhanced derivatized glycidol signals in glycidol-free samples, confirming partial conversion of 3-MCPD to glycidol under basic conditions. This side reaction occurred to different extents depending on temperature (it was observed at both 22 °C and 60 °C, though reaction rates differed). Consequently, maintaining controlled moderate pH is essential to ensure selective derivatization of native glycidol while preventing formation from 2-MCPD and 3-MCPD.

**Figure 4:**
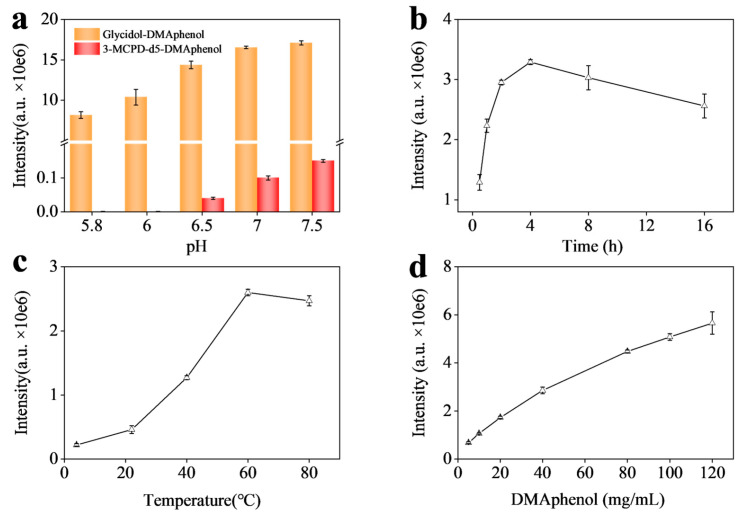
Optimization for derivatization reaction. (**a**) Signal response of glycidol and 3-MCPD-d5 after derivatization at different pH, temperature: 60 °C, DMAphenol: 70 mg/mL, 50 μL, reaction time 4 h; (**b**) Signal response of glycidol after derivatization for different time, pH 6.5 (0.1 M PB buffer), temperature: 60 °C, DMAphenol: 70 mg/mL, 50 μL; (**c**) at different temperature, pH 6.5 (0.1 M PB buffer), DMAphenol: 70 mg/mL, 50 μL, reaction time 4 h; (**d**) and different concentration of DMAphenol, pH 6.5 (0.1 M PB buffer), temperature: 60 °C, reaction time 4 h.

To establish optimal derivatization pH, glycidol-d5 and 3-MCPD-d5 were employed as isotopic internal standards during systematic buffer parametric variation. The glycidol-d5 served to track derivatization efficiency for the target analyte, while 3-MCPD-d5 allowed us to monitor any glycidol-d5 that would form from a chloropropanediol. Figure 4a illustrates the effect of pH on derivatization efficiency and specificity. As the pH was increased from acidic towards neutral, the yield of derivatized glycidol (d5) increased, indicating faster or more complete reaction of glycidol with the reagent. However, above pH 6.5, a noticeable signal for glycidol-d5 began to appear in samples containing 3-MCPD-d5 (but no glycidol originally), demonstrating that 3-MCPD was converting to glycidol under those conditions. By pH 7, this interference became significant. We found that pH 6.5 was the ideal compromise: at pH 6.5, the derivatization of glycidol was efficient (near-maximal product signal) while the conversion of 3-MCPD to glycidol was minimal (Figure 4a). Therefore, pH 6.5 was selected as the buffer condition for all subsequent derivatization reactions. This pH control ensures that our method targets only genuine glycidol and not glycidol artificially generated from other contaminants, thereby upholding the accuracy of the analysis.

Beyond pH optimization, derivatization conditions were systematically refined for reaction duration, temperature, and reagent concentration. The temporal influence on derivative yield is presented in Figure 4b. The response increased as the reaction time was extended, plateauing at around 4 h. Little additional yield was obtained beyond 4 h, so we chose 4 h as the derivatization period to ensure completeness while keeping the overall analysis time reasonable. Although the derivatization time may seem long versus other reactions, it does not mean optimized parameters for high yield are strictly required in practice. According to our experience, it can be reduced to under 1 h at room temperature (22 °C) while maintaining the expected 1 μg/kg sensitivity, as >90% derivatives is produced compared to the maximum amount generated in 4 h, and isotope standards could compensate for inconsistencies, making the process mild and rapid. Figure 4c presents the effect of temperature: raising the reaction temperature from 25 °C to 60 °C improved the derivatization efficiency significantly, but further increase to 80 °C did not produce much higher signal and in fact led to slight degradation of the product (as evidenced by a minor decrease in signal, likely due to side reactions or thermal instability at prolonged 80 °C). Thus, 60 °C was deemed the optimal temperature, providing a good balance between reaction rate and stability of the derivative. Finally, the concentration of DMAphenol was optimized (Figure 4d). We observed that increasing the reagent concentration up to approximately 100 mg/mL resulted in a proportional increase in derivative signal. Beyond 100 mg/mL, the signal gains were marginal, indicating that the reaction was already saturated or limited by glycidol amount. Moreover, excessive concentration of the derivatizing agent also led to an increase in the standard deviation of the derivatization experiments, while potentially introducing an excess of reagent that could contribute to elevated background or matrix effect. Consequently, 100 mg/mL was selected as the working concentration of the derivatization reagent. Under the optimized derivatization conditions (pH 6.5, 60 °C, 4 h, 100 mg/mL reagent), the derivatized glycidol exhibited stable and reproducible LC-MS/MS responses. Stability tests under various stress conditions (Figure S7) confirmed that the derivative remained intact after exposure to different temperatures (25 °C vs. 60 °C) and across a range of dilute acid or base concentrations (0.001–0.1 M HCl or NaOH), with no significant signal variation. This robustness is advantageous for practical applications, demonstrating that the derivatized samples can withstand minor variations in handling and storage without compromising quantification accuracy. Overall, by optimizing these parameters, we significantly enhanced the sensitivity and reliability of glycidol detection while preventing artifactual interference, which is crucial for a complex food like soy sauce.

### 3.3. Pretreatment Process Optimization

#### 3.3.1. Optimization of Sorbent Selection and Elution

Soy sauce presents a challenging matrix for trace analysis due to its rich content of organic compounds and colloidal matter. To achieve accurate glycidol determination, effective sample cleanup is required to remove or reduce matrix components that could interfere with derivatization or detection. We explored several pretreatment strategies to extract glycidol from soy sauce while minimizing losses and interferences.

Initial sample preparation employed dispersive solid-phase extraction (dSPE), whereby a 5:2 (*w*/*w*) adsorbent blend of PestCarb and C18-bonded silica (octadecylsilane) was introduced directly into soy sauce matrices to absorb pigments and hydrophobic interferents. Following vortex mixing and sequential centrifugation (2500 rpm × 10 min × 2), the supernatant was filtered through 0.22 μm PTFE membranes prior to derivatization. This method, however, did not yield a clear solution—the supernatant remained dark and somewhat turbid (Figure S5). The color indicated that pigments were not fully removed, and more importantly, the subsequent derivatization efficiency was poor. The analyte signal was low, probably due to ongoing matrix interference or adsorption of glycidol onto the dispersed sorbents, resulting in an average recovery rate of only 72.2%. Thus, the simple “mix-and-spin” approach was insufficient for this matrix.

We then evaluated conventional SPE cartridges packed with specific sorbents. Two types were tested: a C18 cartridge and a graphitized carbon black (PestCarb-GCB) cartridge. The rationale was that a non-polar C18 sorbent might allow the highly polar glycidol to pass through unretained (eluting with the aqueous phase) while retaining hydrophobic matrix constituents (such as soy sauce pigments and oils). In contrast, a carbon-based sorbent might strongly retain various organic molecules, both polar and non-polar, possibly including glycidol. The low recovery observed with the C18 sorbent can be attributed to this polarity mismatch. Glycidol, as a small polar molecule containing an epoxide group, exhibits minimal affinity for the hydrophobic C18 stationary phase, resulting in poor retention during sample loading and washing steps. Consequently, a substantial portion of glycidol is lost in the flow-through fraction before elution, leading to the observed low recovery. C18 cartridge purification yielded eluates that retained significant pigmentation (Figure S6), more critically, these pigmented eluates led to poor derivatization efficiency in subsequent steps. This suggests that a substantial amount of polar, colored impurities (e.g., sugars, amino acids) were not retained by the C18 sorbent. These co-eluted impurities likely competed with or interfered with the derivatization reaction, resulting in an average recovery rate of only 10.3%. On the other hand, the GCB cartridge, yielded a much less colored extract (Figure S6), indicating effective retention of pigments. GCB is of relatively high cost for single-use in routine high-throughput analysis, compared to the more cost-effective Carbon Yarn alternative.

Considering these results, we sought a more cost-effective yet efficient cleanup strategy. Activated carbon emerged as a suitable candidate: it has a high surface area, broad-spectrum adsorptive power, and is inexpensive. Activated carbon is known for removing organic impurities (e.g., pigments, phenolics) and has been utilized in food processing for decolorization and purification. Although data on the interaction of glycidol with activated carbon were not available, we anticipated that glycidol, being a small polar molecule, might be less strongly adsorbed than larger aromatic compounds, especially in the presence of water. Moreover, using activated carbon aligns with green chemistry principles, as it is a naturally derived, low-cost material with minimal environmental impact [30,31].

**Figure 5:**
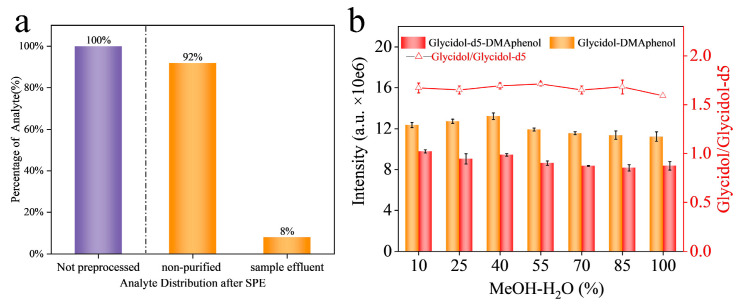
Retention and elution of glycidol on activated carbon cartridge. (**a**) Comparison of the glycidol signal intensity in the non-purified soy sauce extract versus that in the sample effluent after SPE treatment., glycidol concentration: 1 μg/mL; (**b**) signal intensity of eluted glycidol and glycidol-d5 from activated carbon column with MeOH-water mixture of different ratios, load amount: 100 ng/mL, glycidol, 100 ng/mL, Glycidol-d5.

We prepared custom SPE cartridges by packing activated carbon powder into empty SPE tubes. The performance of these activated carbon cartridges was then assessed. When soy sauce was passed through the activated carbon SPE, it became much lighter in color or even nearly colorless (Figure S6), indicating that most pigments and macromolecules were retained on the carbon. To evaluate whether glycidol was being retained by the carbon, we conducted a retention test. A soy sauce sample spiked with a high concentration of glycidol (1 μg/mL) was loaded onto the activated carbon column. We quantified the SPE retention efficiency by comparing the analyte recovery in the flow-through fraction to that in the eluted fraction, with the non-purified extract (Figure 5a). Approximately 92% of glycidol was retained on the activated carbon, demonstrating strong adsorption under the loading conditions. This high retention confirms that the activated carbon effectively captures glycidol while allowing pigments and other matrix components to be retained or washed away.

Next, we optimized the elution step to recover the adsorbed glycidol from the activated carbon. We tested a series of MeOH–water mixtures as eluent, with MeOH content ranging from 10% to 100% (*v*/*v*). Soy sauce samples spiked with 100 ng/mL glycidol were loaded, and then each cartridge was eluted with 2 mL of a given solvent mixture. To account for any variations in derivatization or detection, a fixed amount of internal standard (glycidol-d5) was added to each eluted sample before derivatization, and the glycidol response was also evaluated through the ratio between external and internal standards. The results (Figure 5b) showed that the signal ratio remained fairly consistent across all eluent compositions tested. This indicates that glycidol was effectively eluted even with a low percentage of MeOH and that increasing MeOH to higher percentages did not significantly increase the recovery of glycidol. In other words, all mixtures from 10% up to 100% MeOH were capable of eluting nearly the same amount of glycidol from the carbon. We infer that the presence of some organic solvent is necessary to disrupt interactions between glycidol and carbon, but beyond a certain point, more MeOH does not substantially improve yield.

However, the choice of eluent affected co-elution of matrix components. Eluates with higher MeOH content tended to have a yellow-brown tint, whereas those with 40–55% MeOH were much lighter. This suggests pure or very strong MeOH strips off more matrix constituents in addition to glycidol, while a moderate MeOH–water mix preferentially elutes glycidol with fewer impurities. Using a moderately polar eluent like 40% MeOH is also beneficial for subsequent LC-MS/MS analysis, as it yields a cleaner extract and is compatible with the initial aqueous mobile phase. Considering these factors, we selected 40% MeOH in water as the optimal elution solvent for the activated carbon SPE cleanup. This provided a high recovery of glycidol while keeping the extract relatively clean.

Overall, our optimized sample preparation method employs a self-packed activated carbon SPE column to capture glycidol and remove interfering substances, followed by elution with 40% MeOH to recover free glycidol for analysis without hydrolysis from its ester form. This approach proved to be both effective and economical.

#### 3.3.2. Analytical Specificity Against 2-MCPD and 3-MCPD Interference

To ensure the specificity of this method, it was crucial to verify that 2-MCPD and 3-MCPD do not produce false glycidol signals under our conditions. We conducted specificity tests by spiking blank soy sauce with high levels of 2-MCPD or 3-MCPD (100 ng/mL each, no glycidol) and processing these samples through the entire SPE, derivatization (pH 6.5), and LC-MS/MS procedure. If 2- or 3-MCPD were partially converted to glycidol during any steps, we would detect a glycidol derivative signal in these spiked samples.

We found no glycidol signal in the presence of 3-MCPD or 2-MCPD at a spiked concentration of 100 ng/mL, which are usually a high contamination in soy sauce samples. It implies that even there are conversion, which would be extremely low (below the limit of the detection method). Therefore, under the optimized derivatization conditions (mildly acidic buffer, no strong base added), neither 3-MCPD nor 2-MCPD (including their ester forms, which do not hydrolyze under these parameters) interferes with the glycidol determination. Besides MCPDs, no other known soy sauce constituents produce the same derivative ion signals as glycidol. This specificity is a key advantage of our method: it eliminates the risk of false positives for glycidol that could arise from related contaminants. By maintaining the derivatization at pH 6.5 and using the selective reagent, the method cleanly differentiates glycidol from other compounds in soy sauce.

#### 3.3.3. Matrix Cleanup Effect

Efficient removal of matrix components in the sample prep stage is reflected in improved detection of the target analyte. We quantitatively evaluated the benefit of the activated carbon SPE cleanup on glycidol detection by comparing the glycidol signal in matrix before vs. after cleanup. A blank soy sauce was split into two portions. One portion was derivatized and analyzed directly (matrix present, no SPE) after spiking with a fixed glycidol concentration (mid-range, 20 ng/mL), and the other portion underwent the full SPE cleanup prior to spiking with the same concentration of glycidol and derivatization. The glycidol peak area in the uncleaned sample was taken as reference, and the peak area after cleanup was compared to this.

Our results showed that the glycidol peak area increased from 7.05 × 10^6^ (without cleanup) to 7.92 × 10^6^ (with cleanup). This is about a 12.3% increase in signal intensity. In other words, matrix suppression effects inherent to untreated soy sauce caused measurable signal attenuation of glycidol. Upon selective removal of interferents via activated carbon SPE, glycidol response recovered to near-theoretical levels. While the observed 12% peak area enhancement appears moderate, such improvements are critical for extending method sensitivity in trace analysis, enabling lower detection limits and enhanced quantification reliability. Furthermore, glycidol signal stability improved significantly following matrix cleanup. Crude extracts exhibited signal fluctuations due to ionization suppression from co-eluting interferents, whereas purified extracts demonstrated enhanced temporal stability and baseline continuity. By implementing the activated carbon SPE step, we ensure that the derivatization reaction proceeds with fewer interferences and that the LC-MS/MS detection can operate in a more matrix-free environment. Ultimately, the matrix cleanup contributes to the robustness of the method, making the glycidol analysis more reliable for routine testing of soy sauce samples.

### 3.4. Method Performance

**Figure 6:**
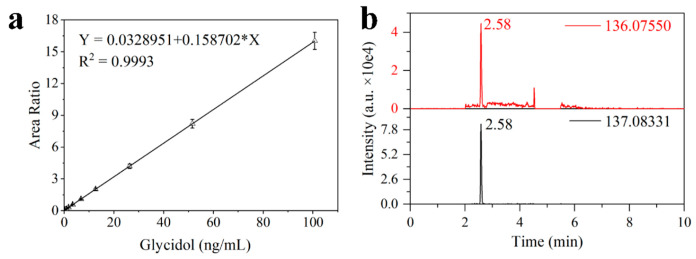
(**a**) Calibration curve of glycidol derivatized with DMAphenol for glycidol quantification, reaction condition: pH 6.5 (0.1 M PB buffer), temperature: 60 °C, DMAphenol: 100 mg/mL, 50 μL, reaction time 4 h; (**b**) Extracted ion chromatogram of glycidol (0.5 ng/mL, LOD) spiked in blank soy sauce after sample preparation and derivatization reaction.

Under optimized conditions, the method performance was rigorously evaluated. The method demonstrated excellent linearity for glycidol in soy sauce over a broad concentration range. Using seven calibrators (1–100 ng/mL), a linear regression of the glycidol/internal standard peak area ratio versus concentration was established, with R^2^ = 0.9993 (Figure 6a). This linearity remained reliable even when extending to lower concentrations (down to 0.1 ng/mL), though points below the LOQ are typically excluded. This high R^2^ value indicates that the internal standard effectively compensated for any variability in sample processing and instrument response, ensuring a consistent proportional relationship. The sensitivity of the method was reflected in the low LOD and LOQ achieved. Based on a signal-to-noise ratio of 3:1, the LOD for glycidol in soy sauce was 0.5 ng/mL (Figure 6b) shows the extracted ion chromatogram at this level). The LOQ, corresponding to S/N = 10:1, was 1 ng/mL. These sensitivity levels are well below the sub-ng/mL concentrations (i.e., sub-ppb, levels) of glycidol detected in real samples, demonstrating that the method is sufficiently sensitive for monitoring trace contamination in soy sauce. Notably, achieving a 0.5 ng/mL LOD in such a complex matrix highlights the advantage of our derivatization and cleanup strategy. In comparison, typical GC-MS methods for glycidol often report higher detection limits due to matrix noise or the need for sample dilution.

Accuracy and precision were evaluated through recovery experiments. Table 2 summarizes the recovery results for glycidol spiked into soy sauce at three levels: 5 ng/mL (average recovery of 102%), 20 ng/mL (100%), and 40 ng/mL (97%). Both the analyte and the internal standard were added to the sample matrix prior to the cleanup procedure. All individual recovery values ranged from 96.6% to 103.1%, within acceptable analytical error. These near-quantitative recoveries indicate minimal loss of glycidol during sample preparation and derivatization, and that the internal standard effectively corrected for minor losses. The precision was also very high, with intra-day RSDs of 1.02%, 1.29%, and 0.51% for low, medium, and high spiked levels, respectively (*n* = 3). These low RSD values demonstrate excellent repeatability. Inter-day precision was also evaluated by analyzing a 20 ng/mL spiked sample over three consecutive days, yielding an average recovery of 85% and an overall RSD of 3.55% (Table S1). The slight decrease in recovery compared to intra-day results (96.6–103.1%) suggests some loss of glycidol over time during storage. This is likely attributable to the inherent reactivity of epoxide ring of glycidol, which may undergo slow ring-opening reactions with nucleophilic components present in the soy sauce matrix (e.g., amines, thiols, or water) during extended storage. we recommend derivatizing soon after extraction/spiking to obtain accurate results. The RSD < 4% confirms good consistency across days. The slightly lower average recovery on different days could be due to the slight degradation of single-day spiked samples for use in different days, small day-to-day variations in calibration or instrument conditions. However, quantification against daily calibration curves (with internal standard) maintained low relative variation across batches. In practical terms, an inter-day RSD of < 4% means the method produces stable results over time, which is crucial for routine monitoring.

Based on the above results, these performance characteristics meet the criteria for quantitative analysis in complex food matrices, indicating the method is well-suited for glycidol surveillance in soy sauce.

### 3.5. Method Comparison

As demonstrated by the preceding validation data, the method developed in this work enables highly sensitive detection of free glycidol in a complex food matrix. It should be noted that analysis of free glycidol across different food matrices, has been scarcely reported. Unlike oils, soy sauce likely contains negligible glycidyl esters, as it undergoes hydrolysis during production. Accordingly, a comparative summary of recently published methods for glycidol determination in various foodstuffs, including both free and its esters forms, which is provided in Table 3. Most current methods rely on derivatization followed by GC–MS analysis. This work is based on LC–MS and employs a straight derivatization procedure conducted in an aqueous phase, which achieves lower detection limit (0.5 ng/mL) and improved recovery. Given the scarcity of directly comparable methods for free glycidol in food matrices, the performance parameters established in this work provide foundation for future studies targeting this contaminant in similar complex, fermented food systems. Moreover, the proposed derivatization method can further be applied in the determination of glycidol esters, or total glycidol content, in different food matrices, after appropriate hydrolysis referencing the official analysis method, which is currently under investigation in our laboratory.

### 3.6. Application to Real Samples

The developed method was applied to measure glycidol in commercially available soy sauces, analyzing 11 different samples (detailed in Section 2.2) using optimized sample preparation, derivatization, and LC-MS/MS procedures. Glycidol was detected in five samples, with concentrations ranging from 1.04 ng/mL to 5.47 ng/mL (Table 4). The highest concentration (5.47 ng/mL) was found in a dark soy sauce, which typically undergoes additional heating or aging, might potentially lead to higher glycidol formation. In our previous research, significantly higher levels of 3-MCPD contamination were also found in dark soy sauce [28], indicating possible shared contamination pathways and their inherent correlation.

The detection of glycidol in soy sauce raises questions about its formation pathways. Based on current knowledge of process contaminant formation, several mechanisms may contribute. Firstly, thermal processing (e.g., pasteurization, concentration) may promote glycidol formation through heat-induced reactions of glycerol or triglycerides with chloride ions [32,33] similar to 3-MCPD formation. The relatively higher level found in dark soy sauce supports this idea. Secondly, trace lipids from raw materials could act as precursors. Thirdly, the correlation between 3-MCPD levels observed in our previous work [28] suggests shared formation pathways. Based on these proposed mechanisms, potential mitigation strategies include optimizing heating conditions (temperature and time), controlling pH during processing, and selecting raw materials with lower precursor content. These mechanisms and strategies are preliminary and require further investigation.

The sample size of eleven was selected as a preliminary set to demonstrate the applicability of the newly developed method across a range of commercially available products, rather than achieving statistical representativeness of the entire soy sauce market. Given that this study primarily focuses on method development and validation, this sample size was deemed sufficient for the initial application and performance evaluation of the analytical method. However, the small sample size limits conclusions about type-based differences, and it could be validated through future large-scale tests of similar samples. Some samples contained glycidol ranging between 1.04–2.08 ng/mL. These levels are substantially lower than the 1000 µg/kg threshold established by EFSA for glycidyl esters in refined oils (approximately 1000 ng/mL if in an aqueous solution). However, chronic low-dose exposure to carcinogens like glycidol may pose cumulative health risks despite compliance with acute regulatory limits. This is the first dataset presenting glycidol contamination across different soy sauce brands and batches. The ubiquitous presence of glycidol suggests that its formation may be an inherent byproduct of the soy sauce production process, likely occurring during heating steps. Manufacturers might consider modifying processing conditions (e.g., reducing excessive heating or adjusting pH) to minimize glycidol generation, although further investigation is required. While there are no current regulatory limits for glycidol in soy sauce, this data provides baseline information for assessing glycidol exposure from condiments. This is the first dataset presenting glycidol contamination across different soy sauce brands and batches.

In terms of method performance, the application to real samples demonstrated that the method is effective for detecting glycidol in hydrolyzed vegetable protein product, such as soy sauce. Internal standard-corrected quantification worked well, and no interference peaks were observed in chromatograms, confirming the specificity of this method. Quality control measures, including mid-level spiking, showed recovery within 5% of expected values, and replicate analysis of one sample yielded an RSD of 3%, indicating good precision. Overall, the detection of glycidol in all tested soy sauces, albeit at low levels, supports the need for routine monitoring. Our LC-MS/MS method provides the necessary sensitivity and selectivity for such monitoring. Future studies could expand the sample size to explore glycidol levels variation in soy sauces from different regions/brands and processing methods, providing further insight into food safety concerns.

**Table 3:** Comparison of the recently published methods on the detection of glycidol in different food matrices.

Matrices	Determination Technique	Derivatization Agent	Solvent	LOD	LOQ	Recoveries	References
Vegetable oil	GC-MS/MS	HFBI (heptafluorobutyric imidazole, glycidol was converted to 3-MBPDE first)	n-hexane	10 μg/kg (expressed as glycidol)	30 μg/kg (expressed as glycidol)	85.4–110%	[34]
Heated tobacco product aerosol	GC-MS/MS	HFBI (heptafluorobutyric imidazole)	Ethyl acetate	-	58.32 ng/cigarette	89–111%	[29]
Edible oils	GC–MS/MS	None	Acetonitrile	10 μg/kg	2–3 μg/kg (free glycidol)	85–115%	[35]
Edible oils and fats	GC–MS	PBA (phenylboronic acid)	NaCl solution	65 μg/kg (glycidyl esters)	-	80–106%	[36]
Fat-rich foodstuffs				15 μg/kg (glycidyl esters)	-	86–90%	
Soy Sauce	HPLC-MS/MS	p-Dimethylaminophenol	Aqueous (NaCl solution)	0.5 μg/kg	1 μg/kg	96.6–103.1%	This work

**Table 4:** Content of glycidol in real soy sauce samples detected using the developed method.

Sample Number	Product Type	Concentration
1	Soy sauce	Not found
2	Soy sauce	Not found
3	Soy sauce	Not found
4	Soy sauce	Not found
5	Soy sauce	2.08 ng/mL
6	Soy sauce	Not found
7	Soy sauce	1.04 ng/mL
8	Soy sauce	1.19 ng/mL
9	Light soy sauce	Not found
10 *	Dark soy sauce	3.05 ng/mL
11 **	Dark soy sauce	5.47 ng/mL

Dilution: 10× (*); 5× (**).

## 4. Conclusions

This study developed a novel derivatization-based LC-MS/MS method for determining free glycidol in soy sauce, a complex food matrix. By incorporating DMAphenol derivatization, optimized pH control, and activated carbon SPE cleanup, the method achieved high specificity, sensitivity, and reliability, detecting glycidol at 0.5 ng/mL and quantifying it from 1 ng/mL upwards across the tested range (to 100 ng/mL) with a linear calibration (R^2^ ≥ 0.9993). The method demonstrated 97–103% recovery and precision better than 5% RSD, highlighting its robustness. Interferences from 2-MCPD and 3-MCPD were eliminated through mild derivatization conditions, ensuring accurate results. Real soy sauce samples confirmed free glycidol presence at low ng/mL levels, emphasizing the importance of a sensitive method. This work addresses a challenge in free glycidol monitoring, offering a tailored approach for soy sauce and potentially other similar non-fatty foods. Although our study employed high-resolution mass spectrometry, this accessible method utilizes readily available reagents and can be easily extended to the most common triple quadrupole instruments. It is therefore well-suited for routine food safety laboratories and furthermore can aid public health surveillance and regulatory efforts. It provides a reliable tool for detecting trace free glycidol without hydrolysis from their ester form, and supports risk management strategies to reduce consumer exposure to harmful substances.

## Figures and Tables

**Table 1:** MS/MS Parameters for the DMAphenol Derivatives of Glycidol and Glycidol-d5.

Compounds	Precursor Ion (*m*/*z*)	Product Ions (*m*/*z*)	CE (eV)	Retention Time (min)
Glycidol-DMAphenol	212.12770	137.08331 ^a^ 136.07550	20 40	2.60
Glycidol-d5-DMAphenol	217.15843	137.08264 ^a^	20	2.57

^a^ indicates that the ion is used for the quantitative analysis of the analyte.

**Table 2:** Spiked recoveries and precision for glycidol in soy sauce (*n* = 3 for each level).

Spiked Level (ng/mL)	Recovery Range (%)	Average Recovery (%)	Intra-Day RSD (%)
5	101.2–103.1	102	1.02
20	98.3–100.9	100	1.29
40	96.6–97.6	97	0.51

## Data Availability

The original contributions presented in the study are included in the article/Supplementary Materials. Further inquiries can be directed to the corresponding authors.

## Supplementary Materials

The following supporting information can be downloaded at: https://www.mdpi.com/article/10.3390/foods15071220/s1, Figure S1. High-resolution mass spectrum of the derivatized product of glycidol-d5 with p-Dimethylaminophenol (500 ng/mL, in methanol, electrospray ionization). Figure S2. Chromatogram of the derivatized glycidol-d5 in soy sauce matrix (0.5 ng/mL, monitored by LC-MS/MS with electrospray ionization). Figure S3. ^13^C NMR spectrum of derivatized product of glycidol with p-Dimethylaminophenol. Figure S4. ^1^H NMR spectrum of derivatized product of glycidol with p-Dimethylaminophenol. Figure S5. Comparison of supernatants afte dSPE cleanup: Soy sauce samples treated with 5:2 (*w*/*w*) PestCarb and C18-bonded silica (octadecylsilane) blend following sequential centrifugation. Figure S6. Appearance of soy sauce samples after passing through SPE cartriadges with different adsorbents. Figure S7. Stability of the derivatized glycidol under various chemical and thermal conditions (10 h treatments). The derivative (1 ppm solution in 40% methanol–water) was exposed to different conditions and then analyzed by LC–MS/MS. A: Control (25 °C, no added acid or base); B: 60 °C with 0.1 M NaOH; C: 60°C with 0.01 M NaOH; D: 60 °C with 0.001 M NaOH; E: 60 °C with 0.1 M HCl; F: 60 °C with 0.01 M HCl; G: 60 °C with 0.001 M HCl. The mass spectrometric response for the glycidol derivative remained consistent across all conditions (A–G), with no significant degradation or new by-product formation. Even in the presence of moderately strong acid or base at elevated temperature, the signal of the derivative and retention time were unchanged, demonstrating its high chemical stability. Table S1. Inter-day recovery of glycidol (2, 5, 20 ng/mL spiked into soy sauce) over three consecutive days (n = 3 replicates per day). The table shows the measured recovery percentage each day, the average recovery, and the relative standard deviation (RSD). The results indicate good method reproducibility across different days.
