# Supplementary material for: Determination of Glycidol in Soy Sauce Using p-Dimethylaminophenol Derivatization Coupled with Liquid Chromatography-Tandem Mass Spectrometry
Source: Foods. 2026 Apr 3;15(7):1220. doi: 10.3390/foods15071220 (PMC13073954; doi:10.3390/foods15071220)
Supplement: Supplementary file 1 [file foods-15-01220-s001.zip › foods-4151310-supplementary.pdf]

The mass spectrometry and NMR data (Figures S1 and S4) corroborate the successful derivatization of glycidol. In particular, the presence of the expected m/z 217 ion for derivatized glycidol-d5 (Figure S1) and its identical fragmentation pattern to the unlabeled derivative confirm that the isotopically labeled internal standard behaves analogously to the target, validating our quantitative approach. The matching retention times of labeled vs. unlabeled derivatives further indicate no isotopic effect on chromatography, meaning glycidol-d5 is an ideal internal standard for this method. The NMR spectra (Figures S3 and S4) provide structural confirmation: all significant proton and carbon signals can be assigned to the proposed structure of the glycidol–aromatic derivative. For instance, the aromatic protons at δ 7.68 and 6.95 ppm (doublets with J = 9.6 Hz) are characteristic of the p-Dimethylaminophenol ring, and the appearance of N-methyl proton signals at δ 3.64 and 3.55 ppm (singlets) confirms the presence of the dimethylamino substituent. Meanwhile, signals in the δ 3.4-5.4 ppm range correspond to the protons on the glycerol backbone (including hydroxyl groups), consistent with ring-opening of glycidol and attachment of the derivatizing agent. These details reinforce that the structure of the derivative is exactly as expected, lending confidence that our detection is accurate for glycidol.

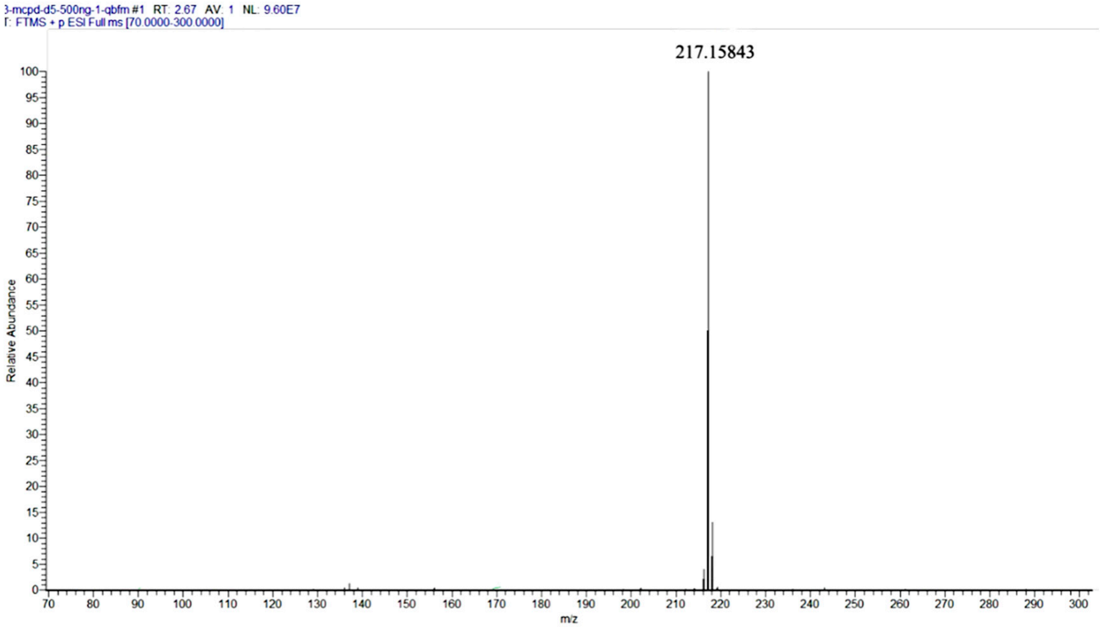


**Figure S1.** High-resolution mass spectrum of the derivatized product of glycidol-d5 with p-Dimethylaminophenol (500 ng/mL, in methanol, electrospray ionization).

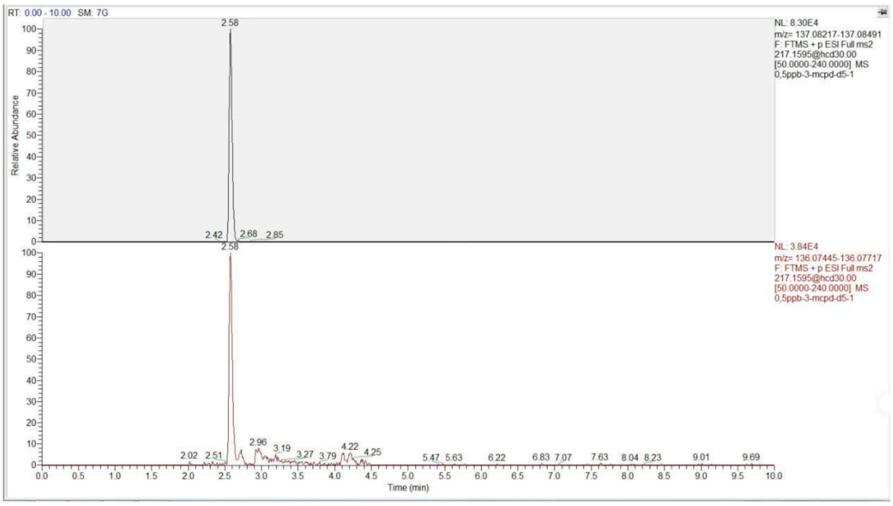


**Figure S2.** Chromatogram of the derivatized glycidol-d5 in soy sauce matrix (0.5 ng/mL, monitored by LC-MS/MS with electrospray ionization).

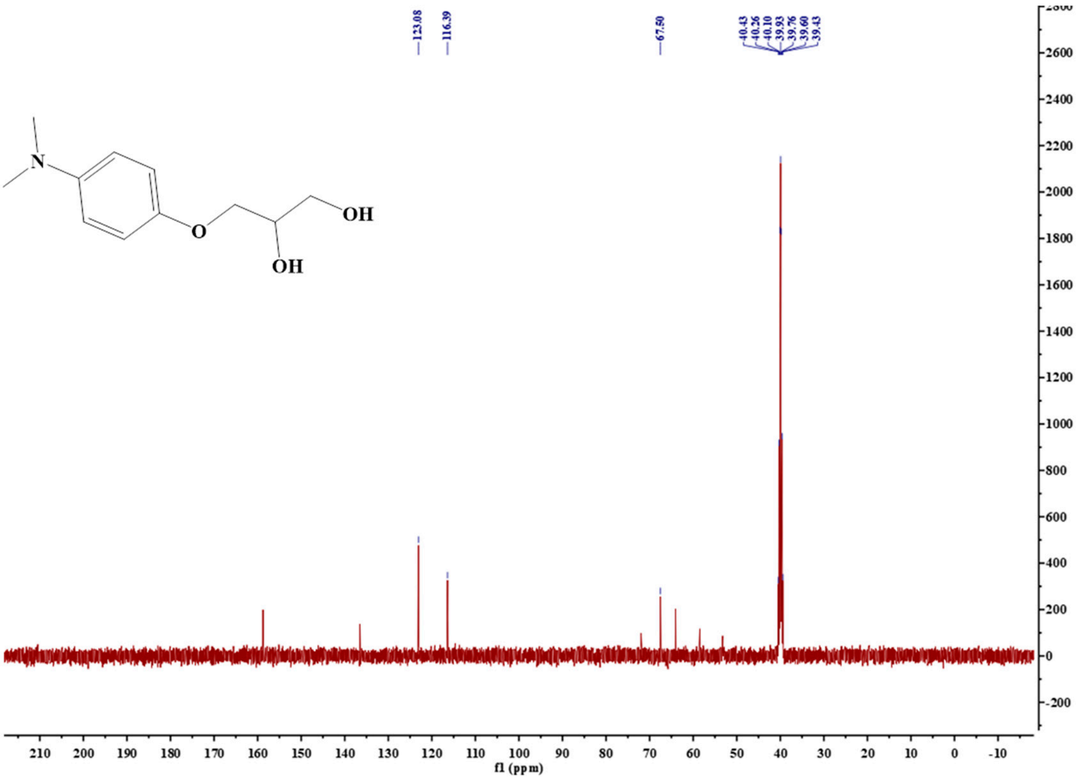


**Figure S3.** $^{13}$C NMR spectrum of derivatized product of glycidol with p-Dimethylaminophenol.

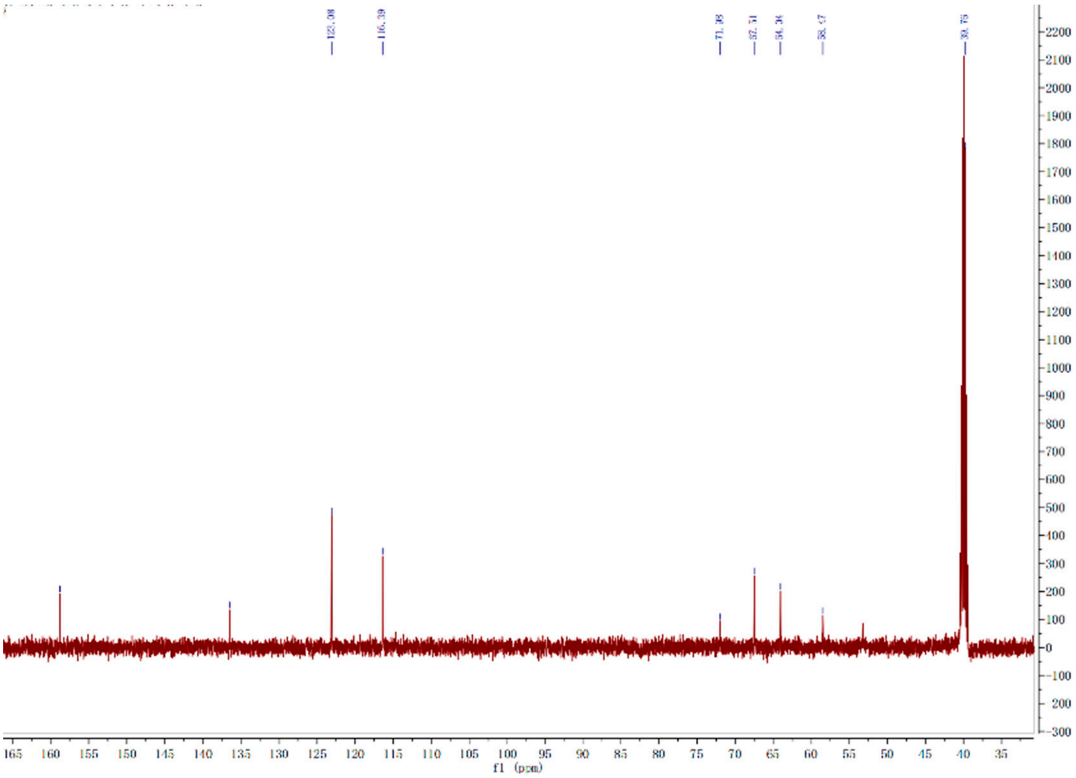


**Figure S4.** $^{1}H$ NMR spectrum of derivatized product of glycidol with p-Dimethylaminophenol.

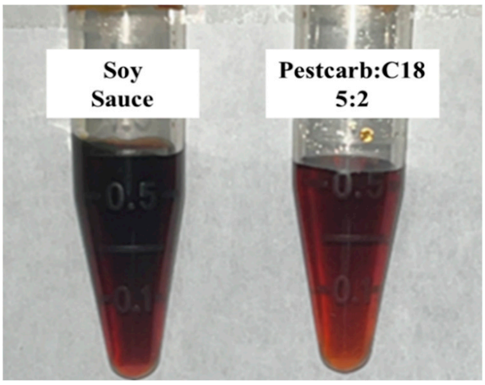


**Figure S5.** Comparison of supernatants afte dSPE cleanup: Soy sauce samples treated with 5:2 (w/w) Pest-Carb and C18-bonded silica (octadecylsilane) blend following sequential centrifugation.

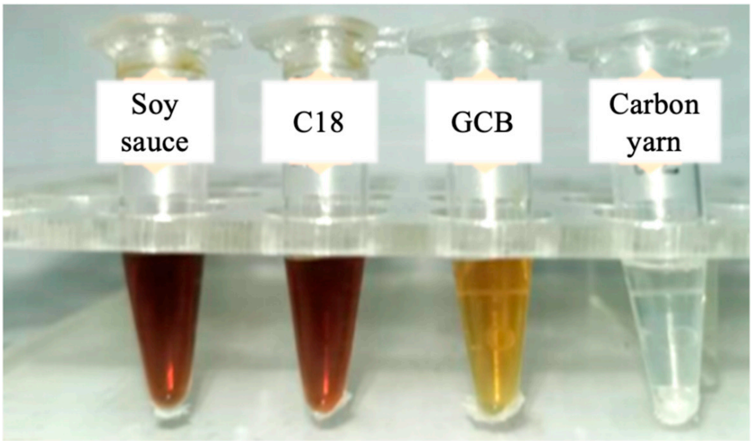


**Figure S6.** Appearance of soy sauce samples after passing through SPE cartriadges with different adsorbents.

**Table S1.** Inter-day recovery of glycidol (2, 5, 20 ng/mL spiked into soy sauce) over three consecutive days (n = 3 replicates per day). The table shows the measured recovery percentage each day, the average recovery, and the relative standard deviation (RSD). The results indicate good method reproducibility across different days.

| Spiked level (ng/mL) | First day recovery(%) | Second day recovery(%) | Third day recovery (%) | Average recovery (%) | RSD (%) |
|---|---|---|---|---|---|
| 2 | 114 | 113 | 110 | 112 | 1.64 |
| 5 | 107 | 101 | 106 | 105 | 3.49 |
| 20 | 83 | 89 | 84 | 85 | 3.55 |

Table S1 quantifies the intermediate precision of the method through inter-day reproducibility assessments. The spiked concentrations of 2 and 5 ng/mL yielded acceptable recovery and precision over three consecutive days. The recoveries of 83-89% for a 20 ng/mL spike on three different days resulted in an average of 85% with RSD 3.55%. The slightly lower than 100% average recovery might be due to small systematic differences on each day (for example, minor pipetting errors, or slight variations in SPE cartridge packing). However, because an internal standard was used and a fresh calibration was prepared each day, the relative consistency is high (RSD = 3.5%). This suggests that while absolute recoveries can vary, the quantification remains reliable day-to-day after calibration adjustment. In a routine lab setting, one could further improve accuracy by including a matrix-matched quality control sample to

correct any bias, but the low RSD indicates such corrections would be minor. Overall, the inter-day data affirm that the method is reproducible over time, an important aspect for a method that might be used regularly for monitoring.

The stability tests (Figure S7) demonstrate that once glycidol is derivatized, the resulting compound is robust under a variety of conditions. The signal intensity of derivative remained stable after incubation at 60°C for 10 h in 0.1 M NaOH or HCl. In practical terms, this means that the derivatized samples can tolerate slightly non-ideal storage or processing conditions (e.g., if a derivatized batch needs to wait before injection, or if the solvent has some residual acidity/alkalinity) without compromising the glycidol quantification. The stability of the derivative in both acidic and basic environments also indicates that any residual salts or buffers from the sample matrix are unlikely to cause decomposition during analysis. This finding is important for method ruggedness, as it shows the derivatization effectively "locks" glycidol into a form that is not easily affected by external conditions.

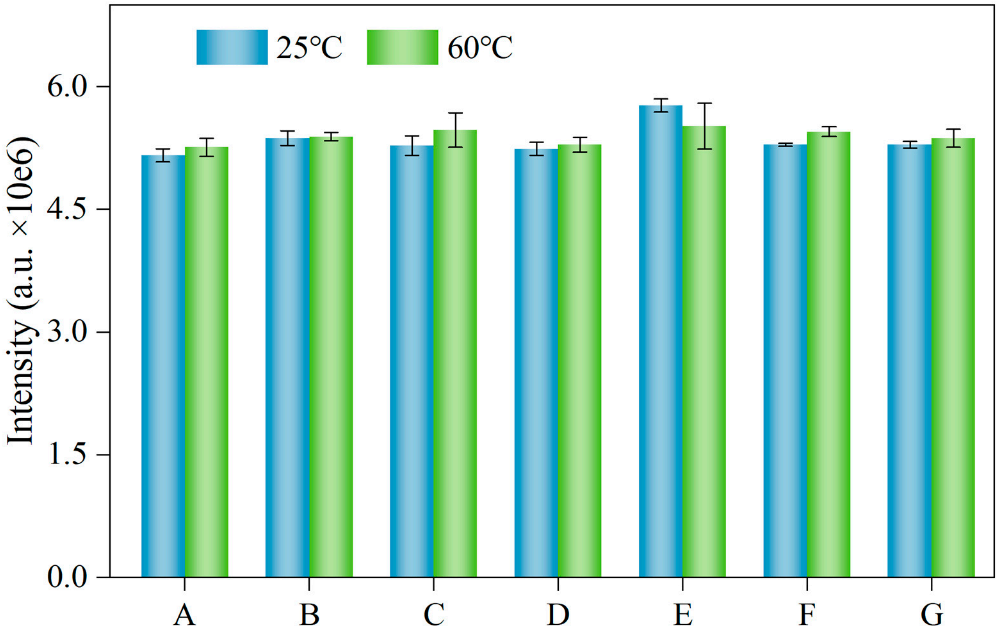


**Figure S7.** Stability of the derivatized glycidol under various chemical and thermal conditions (10 h treatments). The derivative (1 ppm solution in 40% methanol–water) was exposed to different conditions and then analyzed by LC–MS/MS. A: Control (25°C, no added acid or base); B: 60°C with 0.1 M NaOH; C:

60°C with 0.01 M NaOH; D: 60°C with 0.001 M NaOH; E: 60°C with 0.1 M HCl; F: 60° C with 0.01 M HCl; G: 60°C with 0.001 M HCl. The mass spectrometric response for the glycidol derivative remained consistent across all conditions (A-G), with no significant degradation or new by-product formation. Even in the presence of moderately strong acid or base at elevated temperature, the signal of the derivative and retention time were unchanged, demonstrating its high chemical stability.

In conclusion, the supporting information data bolster the confidence in our analytical method. The MS and NMR results confirm we are measuring the correct compound (derivatized glycidol), the stability tests prove the derivative is hardy (which contributes to consistent measurements), and the inter-day study shows the method yields repeatable results across different runs. These additional pieces of evidence complement the main manuscript by addressing potential questions about identity, stability, and reproducibility of the analyte under our method conditions.
